# Diagnostic Overshadowing and the Unseen Spectrum: A Narrative Review of Rare Complications in Sickle Cell Disease

**DOI:** 10.3390/clinpract15090156

**Published:** 2025-08-27

**Authors:** Abdulrahman Nasiri, Manal Alshammari, Reem Alkharras, Albaraa Madkhali, Mostafa F. Mohammed Saleh, Hazza Alzahrani

**Affiliations:** 1 Department of Internal Medicine, College of Medicine, Imam Mohammad Ibn Saud Islamic University (IMSIU), Riyadh 11432, Saudi Arabia; 2 Hematology Department, King Salman Specialist Hospital, Hail 55471, Saudi Arabia; 3 Department of Internal Medicine, Security Forces Hospital, Riyadh 11481, Saudi Arabia; 4 Department of Internal Medicine, Armed Forces Hospital, Jizan 45142, Saudi Arabia; 5 Hematology Department, King Faisal Specialist Hospital & Research Centre, Riyadh 12713, Saudi Arabia

**Keywords:** sickle cell disease, diagnostic overshadowing, rare complications, fat embolism syndrome, acute leukemia, intrahepatic cholestasis, hemophagocytic lymphohistiocytosis, spontaneous epidural hematoma, extramedullary hematopoiesis, multiorgan failure syndrome

## Abstract

Sickle cell disease (SCD) is a hereditary hemoglobin disorder characterized by chronic hemolysis and recurrent vaso-occlusive crises, leading to a wide spectrum of complications. While common SCD manifestations have well-established management protocols, rare and atypical complications pose significant diagnostic and therapeutic challenges. A critical barrier is diagnostic overshadowing, where common SCD symptoms (pain, fever, respiratory distress) mask infrequent but life-threatening conditions, resulting in delayed recognition and suboptimal outcomes. This narrative review synthesizes the literature from 2000–2025 on rare SCD complications, including atypical neurological events (e.g., spontaneous epidural or subdural hematoma, central retinal artery occlusion, cerebral arteriovenous malformations, posterior reversible encephalopathy syndrome), uncommon hematologic syndromes (acute leukemia, extramedullary hematopoiesis in unusual sites, hemophagocytic lymphohistiocytosis), severe cardiopulmonary emergencies (acute multiorgan failure and fat embolism syndromes), unusual hepatic crises (acute hepatic sequestration, intrahepatic cholestasis), and others (e.g., compartment syndrome). Key insights underscore the need for high clinical suspicion and prompt use of advanced diagnostics (e.g., MRI, specialized laboratory tests) when patients present with atypical or disproportionate symptoms. Clinical implications: Heightening clinician awareness of these rare complications and implementing structured diagnostic strategies can facilitate earlier intervention, improving outcomes and reducing the high morbidity and mortality associated with these infrequent but severe events.

## 1. Introduction

Sickle cell disease (SCD) is a group of inherited hemoglobin disorders characterized by chronic hemolysis and recurrent vaso-occlusion, leading to widespread organ damage and significant morbidity and mortality. The pathophysiology centers on the polymerization of deoxygenated sickle hemoglobin (HbS), causing erythrocytes to deform into a sickle shape. These rigid cells obstruct the blood flow in small vessels, precipitating episodes of acute pain, termed vaso-occlusive crises (VOCs), which are the hallmark clinical presentation. Beyond VOCs, SCD is associated with numerous acute and chronic complications that affect nearly every organ system, including acute chest syndrome, stroke, pulmonary hypertension, chronic kidney disease, and avascular necrosis [1]. Pregnancy also poses unique risks for women with SCD, including hypertensive disorders and thromboembolism, although rarer obstetric complications can also occur [2]. Severe infections represent a significant cause of morbidity and mortality in SCD, with an increased risk of life-threatening infectious complications [3].

While the management of common SCD complications is relatively well-established, clinicians frequently encounter diagnostic and therapeutic challenges posed by rarer, atypical presentations. A significant issue in the care of patients with SCD is diagnostic overshadowing, defined as the tendency for common SCD-related symptoms to dominate the clinical picture and thereby mask co-existing uncommon complications. In other words, clinicians may attribute new or severe symptoms solely to a typical sickle crisis, overlooking a concurrent rare condition. This phenomenon leads to delayed diagnoses and suboptimal management of rare events, particularly in acute crisis settings where rapid assessment is critical. This review highlights cases where SCD has presented unusually, such as the de novo diagnosis of an older adult during an unrelated illness or decompensated cirrhosis as a first presentation [4].

Recognition of rare complications in SCD is not merely an academic exercise, it has direct clinical importance. These infrequent manifestations, though uncommon, contribute disproportionately to SCD-related mortality and morbidity. For instance, fat embolism syndrome (FES) in SCD can be rapidly fatal, with reported mortality rates around 46% [5], and sickle cell intrahepatic cholestasis, although extremely rare, is often lethal. Delayed or missed diagnosis of such entities can lead to preventable deaths. Furthermore, rare complications may be underdiagnosed in regions with limited resources or in populations with reduced access to specialty care, which contributes to health disparities. The majority of patients with SCD worldwide reside in sub-Saharan Africa and other resource-limited settings, where the diagnostic infrastructure may be insufficient to detect uncommon complications, which exacerbates outcome gaps. Emphasizing the spectrum of SCD complications, including the rare and atypical, is therefore essential to improve equity in care and to reduce avoidable mortality [6]

Awareness regarding the spectrum of rare complications in SCD remains a crucial gap among many clinicians, including hematologists, internists, and frontline emergency and hospital medicine physicians. The evidence base for these complications is primarily derived from individual case reports and small case series, which reflects their infrequent occurrence but highlights their clinical significance.

This narrative review aims to synthesize the current knowledge on rare and unusual complications of SCD that has been published over the past two decades. Drawing upon a comprehensive literature search of case reports, case series, and reviews, we provide a clinically relevant resource detailing the presentation, diagnostic challenges, and pathophysiology of these less common manifestations, as well as recommended investigations and management strategies. By consolidating these findings, we seek to enhance clinician awareness and facilitate timely recognition and appropriate management of rare complications during acute crises and routine care in patients with SCD.

## 2. Methods

We performed a systematic narrative review to synthesize the current knowledge on rare and atypical complications of sickle cell disease (SCD), focusing on the impact of diagnostic overshadowing. Our approach followed the PRISMA recommendations adapted for narrative synthesis.

### 2.1. Search Strategy

A comprehensive search of PubMed, Embase, and Scopus databases was conducted to identify the relevant literature published between 1 January 2000 and 30 April 2025. The search combined Medical Subject Headings (MeSH) and free-text terms related to SCD and rare clinical manifestations. The key search terms included disease-specific phrases such as “Sickle Cell Disease”, “Sickle Cell Anemia”, and “Sickle Cell” combined with qualifiers indicating rarity and clinical complexity such as “rare”, “unusual”, “atypical”, and “complicated” and linked with descriptors of clinical presentation including “complication”, “manifestation”, “syndrome”, “event”, and “presentation”. To focus on relevant evidence, the search included publication types like “case report”, “case series”, “literature review”, and “review”. Furthermore, specific rare complications known from preliminary investigations were included to maximize sensitivity. These encompassed terms such as “epidural hematoma”, “subdural hematoma”, “spinal infarction”, “cerebral arteriovenous malformation”, “pulmonary arteriovenous malformation”, “hemophagocytic lymphohistiocytosis”, “fat embolism syndrome”, “hepatic sequestration”, “intrahepatic cholestasis”, “adrenal hemorrhage”, “renal medullary carcinoma”, “compartment syndrome”, “posterior reversible encephalopathy syndrome”, “hyperhemolysis syndrome”, and “delayed hemolytic transfusion reaction”

### 2.2. Selection Criteria

Two independent reviewers screened titles and abstracts for relevance, identifying studies that reported rare or unusual clinical manifestations of SCD, particularly those highlighting diagnostic challenges. We included peer-reviewed case reports, case series, and narrative or systematic reviews published in English between 2000 and 2025. There were no age restrictions, so both pediatric and adult patient reports were included.

Exclusion criteria were editorials, conference abstracts without full text, and studies lacking clinical detail. Articles not in English were excluded.

### 2.3. Data Extraction and Quality Assessment

Relevant articles underwent full-text review. Data extracted included clinical presentation, diagnostic methods, pathophysiology, treatment, and outcomes. We appraised the quality of the evidence using a modified Oxford Centre for Evidence-Based Medicine scale, acknowledging the predominance of low-level evidence inherent to case reports.

### 2.4. Synthesis Approach

Due to heterogeneity and predominance of descriptive data, we performed a qualitative narrative synthesis organized by organ system and complication type. We highlighted common diagnostic pitfalls and the phenomenon of diagnostic overshadowing, supported by relevant clinical examples.

## 3. Results

**Figure 1:**
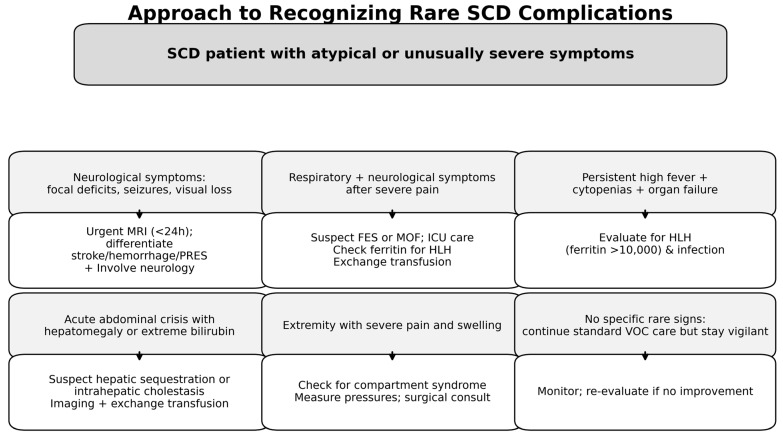
Diagnostic approach to rare complications in sickle cell disease.

Based on the literature search and inclusion criteria, we identified a range of rare and unusual SCD complications. For clarity, the results are organized by organ system or syndrome type. Each subsection describes key clinical findings, diagnostic challenges (with attention to overshadowing by common SCD symptoms), the pathophysiology of the case, recommended investigations, management strategies, and any known preventive considerations for that complication. A summary algorithm for recognizing rare complications in SCD is provided (Figure 1), and Table 1 summarizes key features and outcomes of the identified complications.

This algorithm outlines a symptom-driven strategy for early recognition of rare but severe complications in sickle cell disease (SCD). It emphasizes prompt imaging and targeted evaluation for posterior reversible encephalopathy syndrome (PRES), fat embolism syndrome (FES), hemophagocytic lymphohistiocytosis (HLH), acute multiorgan failure (MOF), hepatic crises, and compartment syndrome. The following abbreviations are used: SCD, sickle cell disease; PRES, posterior reversible encephalopathy syndrome; FES, fat embolism syndrome; HLH, hemophagocytic lymphohistiocytosis; MOF, multiorgan failure; CRAO, central retinal artery occlusion; EMH, extramedullary hematopoiesis; VOC, vaso-occlusive crisis.

### 3.1. Neurological Complications

Beyond common manifestations like overt stroke and silent cerebral infarcts, SCD can present with extremely rare neurological events that often mimic or occur alongside VOC. Heightened suspicion is crucial in patients presenting with new or atypical neurological symptoms during crisis [7].

#### 3.1.1. Spontaneous Epidural and Subdural Hematoma

- **Definition:** non-traumatic bleeding into the epidural or subdural space surrounding the spinal cord or brain;
- **Key case findings:** Several case reports describe non-traumatic presentations of spontaneous epidural hematoma in young adults and children with SCD, frequently amidst sickle crises, causing sudden onset of back pain, radicular pain, or neurological deficits [8]. Spontaneous subdural hemorrhage has also been reported as mimicking dural sinus thrombosis in an adolescent [9] and presenting as chronic bilateral subdural hematoma in a young child [10]. Unilateral papilledema has revealed a subdural hematoma in an adult patient [11];
- **Diagnostic challenges:** A sudden onset of back pain, radicular pain, or neurological deficits (weakness, sensory changes, bowel/bladder dysfunction) can be attributed solely to VOC, which can delay appropriate imaging [7,8]. Subdural bleeds can also present with non-specific headaches or neurological changes that might be overshadowed by concurrent pain or anemia symptoms;
- **Pathophysiologic rationale:** Hypothesized mechanisms of this symptom include skull or vertebral bone infarction, altered skull/vertebral anatomy from extramedullary hematopoiesis (EMH), and venous congestion during crises [7,8]. Subdural bleeds may also relate to vascular fragility or altered coagulation in SCD [9];
- **Recommended diagnostic tests:** Urgent neuroimaging is essential. An MRI of the spine or brain should be obtained immediately (ideally within hours of symptom onset) to confirm an epidural or subdural hematoma and delineate its extent. If intracranial pathology is suspected, brain MRI is preferred; for isolated spinal symptoms, spinal MRI is indicated (in acute settings where MRI is not instantly available, CT can be a first step, but MRI provides superior detail of spinal/soft tissue bleeding) [8].
- **Management strategies and outcomes:** Prompt neurosurgical evaluation for decompression is often required. Laminectomy or craniotomy to evacuate the hematoma can lead to good neurological recovery if performed early. Supportive care (hydration, transfusion to reduce sickling, blood pressure control) is also important. The outcomes are favorable when intervention is timely, but delays can result in permanent paralysis or other deficits [7,8,9,10,11];
- **Prevention:** There are no specific measures to prevent spontaneous hemorrhages, but optimal SCD management (e.g., maintaining hemoglobin levels, avoiding extreme anemia or dehydration) might reduce precipitating factors such as severe bone infarction. Additionally, clinicians should promptly investigate any atypical neurological pain in SCD rather than attributing it solely to VOC;
- **Clinical takeaway:** Unexplained or disproportionately severe spinal pain or neurological deficits in a patient with SCD mandate urgent neuroimaging to rule out epidural hematoma. Atypical headaches or neurological signs should prompt consideration of subdural hemorrhage.

#### 3.1.2. Brain and Spinal Infarcts (Atypical Presentation) and Cerebral Arteriovenous Malformations

- **Definition:** ischemic injury to the brain or spinal cord tissue due to vascular occlusion, presenting in unusual locations or contexts beyond typical stroke, or rare vascular malformations;
- **Key case findings:** A retrospective review of the spinal pathology in SCD highlights spinal cord infarction as a significant complication [12]. A case report details an autopsy-confirmed spinal cord infarction in a patient with sickle cell anemia [13]. Another case describes an anterior spinal infarct in a 19-year-old man [14]. In one dramatic case, a 32-year-old woman with otherwise stable SCD developed septic shock and acute multiorgan failure (MOF) during a severe crisis, with rapid neurological decline and death; it was postulated that atypical patterns of cerebral infarction occurred in the context of MOF [15]. The development of de novo cerebral arteriovenous malformation (AVM) in a child with SCD and moyamoya arteriopathy has also been reported, with the rarity and lack of an established correlation between cerebral AVMs and SCD being noted [16];
- **Diagnostic challenges:** Neurological impairment may be overshadowed by systemic symptoms of severe crisis or MOF [15]. Recognizing infarcts in unusual locations (e.g., spinal cord, atypical brain regions) or concurrently with widespread organ failure is challenging. Cerebral AVMs may be asymptomatic until they hemorrhage or cause seizures, and in SCD these symptoms might initially be thought to result from more common causes (stroke or meningitis);
- **Pathophysiologic rationale:** Vaso-occlusion, potentially exacerbated by systemic inflammation, infection (septic shock), or hypercoagulability (e.g., associated antiphospholipid antibodies), contributes to vessel occlusion and tissue infarction [15]. The development of AVMs in SCD is poorly understood but may relate to chronic vascular remodeling or angiogenesis in response to ischemia, particularly in the setting of moyamoya [16];
- **Recommended diagnostic tests:** Any severe or atypical neurological deficit in SCD warrants thorough imaging. Brain MRI (with vascular sequences) and spinal MRI should be performed to localize infarcts. If an AVM is suspected or MRI suggests an abnormal tangle of vessels, cerebral angiography is the gold standard for diagnosis. In patients with unusual presentations (e.g., during sepsis or MOF), also evaluate for precipitants: blood cultures, inflammatory markers, and coagulation studies including antiphospholipid antibodies may be informative;
- **Management strategies and outcomes:** Management of infarcts involves acute stroke protocols (exchange transfusion to reduce the HbS < 30%, hydration, oxygen, treatment of any infection) and supportive ICU care for MOF. Blood exchange was attempted in one severe MOF case but the patient succumbed [15]. Maintaining the HbS below 30% through exchange transfusion is suggested to reduce thrombotic complications. Management of AVMs is complex and may involve surgical resection, endovascular embolization, or radiosurgery [16];
- **Prevention:** Standard SCD stroke prevention strategies (e.g., transcranial Doppler screening in children, prophylactic transfusions for high-risk patients) help prevent common strokes and may incidentally prevent some atypical infarcts. Keeping the HbS percentage low during high-risk periods (e.g., perioperatively or during severe illness) might reduce the infarct risk [17]. There is no known prevention for AVMs, but controlling the severity of SCD over the long term might mitigate the chronic vascular stress that potentially contributes to such anomalies;
- **Clinical takeaway:** Severe or atypical neurological deficits in a patient with SCD, even in the context of systemic illness, require imaging to assess for infarction. AVMs are rare but important to consider in those with seizures or hemorrhage. Associated conditions like infection or hypercoagulability should be considered.

#### 3.1.3. Central Retinal Artery Occlusion (CRAO)

- **Definition:** acute occlusion of the central retinal artery (or its branches), leading to sudden, painless monocular vision loss;
- **Key case findings:** While proliferative sickle retinopathy and peripheral vascular occlusions are common, CRAO is a rare and devastating complication, often presenting with sudden, painless loss of vision. A case report details concurrent bilateral CRAO during an SCD crisis that was managed with erythrocytapheresis [18];
- **Diagnostic challenges:** In a patient with SCD, acute vision loss might initially be attributed to more common causes like vitreous hemorrhage, retinal detachment, or even an ocular manifestation of severe anemia. Distinguishing CRAO requires prompt ophthalmologic examination, which can be delayed if the patient’s focus (and the clinicians’) is on systemic pain or other crisis symptoms. Additionally, if only one eye is affected, patients in severe pain may not immediately report vision changes;
- **Pathophysiologic rationale:** Sickled erythrocytes can cause thrombotic occlusion of the central retinal artery or its branches, especially under conditions of severe hypoxemia or acidosis during crises. This leads to retinal ischemia. Unlike peripheral sickle retinopathy, CRAO involves a large vessel supplying the inner retina, analogous to a stroke in the eye;
- **Recommended diagnostic tests:** Immediate ophthalmologic evaluation is critical. Fundoscopic examination classically shows a pale retina with a “cherry-red spot” at the macula in CRAO. Optical coherence tomography (OCT) can confirm retinal swelling, and fluorescein angiography can localize the occlusion;
- **Management strategies and outcomes:** CRAO is an ophthalmologic emergency. Interventions (though often of limited success) aim to restore retinal blood flow: ocular massage, anterior chamber paracentesis, and medications to lower intraocular pressure can be tried [18]. Systemic thrombolysis has also been reported in a case treated with IV alteplase and exchange transfusion [19];
- **Prevention:** The primary method of preventing CRAO in SCD is maintaining overall good control of the disease to prevent extreme sickling episodes. Regular ophthalmologic screening in SCD can detect early proliferative changes but will not predict CRAO. Avoidance of risk factors like uncontrolled hypertension or hyperviscosity (if on transfusions) may theoretically help [20];
- **Clinical Takeaway:** Sudden, painless vision loss in patient with SCD is a medical emergency requiring urgent ophthalmologic consultation to rule out CRAO.

#### 3.1.4. Posterior Reversible Encephalopathy Syndrome (PRES)

- **Definition:** a neuro-radiological syndrome characterized by vasogenic edema, predominantly in the posterior white matter, and often associated with hypertension.
- **Key case findings:** PRES has been increasingly recognized as a rare complication in SCD, particularly in the setting of hypertension during crisis, although it can also occur normotensively. Published cases include both pediatric and adult patients with SCD developing PRES [21]. A case series and literature review specifically address PRES in adult patients with SCD [22]. PRES has also been reported in sickle-beta-thalassemia [23]. Common features in reported cases are headaches, seizures, visual disturbances, and encephalopathy during or following a VOC or hemolytic crisis;
- **Diagnostic challenges:** Symptoms of PRES (headache, visual disturbances, seizures, altered mental status) overlap with other neurological complications or metabolic derangements in SCD crisis. Distinguishing PRES requires neuroimaging; however, if clinicians focus only on SCD-related causes (e.g., stroke), they might overlook the pattern that is characteristic of PRES. In patients with SCD, pain and distress can elevate blood pressure, potentially precipitating PRES, but the link may not be immediately recognized. Diagnostic overshadowing can occur if providers assume seizures or visual changes are due to known stroke risk without considering PRES, which has a different treatment focus (blood pressure and symptom control);
- **Pathophysiologic rationale:** The exact mechanism of PRES involves failure of cerebral autoregulation, often due to acute hypertension or endothelial dysfunction, leading to hyperperfusion and leakage of fluid into the brain’s interstitium (vasogenic edema). In SCD, severe anemia, high circulating cell-free hemoglobin, and inflammation may contribute to endothelial injury. Rapid swings in blood pressure during crises, or severe systemic inflammation (even without hypertension), can precipitate PRES. Notably, patients with SCD may have baseline endothelial activation, which possibly lowers the threshold for PRES [21,22];
- **Recommended diagnostic tests:** Brain MRI is the diagnostic modality of choice for PRES. It classically shows T2/FLAIR hyperintensities (edema) in the posterior occipital and parietal lobes, which are often symmetrical. Diffusion-weighted imaging helps distinguish vasogenic edema (PRES) from cytotoxic edema (infarction). In suspected PRES, blood pressure measurement and control are imperative; labs should include renal function (to check for hypertensive injury) and consideration of other causes (e.g., eclampsia in pregnant patients). Importantly, in any patient with SCD with neurologic symptoms, an MRI should be obtained as soon as possible, ideally within 24 h, to differentiate PRES from acute ischemic stroke or hemorrhage, as their management differs;
- **Management strategies and outcomes:** The cornerstone of PRES management is aggressive treatment of the underlying cause: typically blood pressure and seizure management. PRES is typically reversible with treatment of the underlying cause [21,22];
- **Prevention:** For patients with SCD who are at risk (e.g., known history of PRES or severe hypertension episodes), careful blood pressure monitoring and control during acute pain episodes is advisable. Avoiding an excessive transfusion volume (to prevent hypertension) and treating pain adequately (to mitigate adrenergic surges) may help. There is no specific prophylactic medication for PRES, but general measures to maintain vascular health (e.g., hydroxyurea to reduce hemolysis and nitric oxide consumption) might theoretically lower the risk [24];
- **Clinical takeaway:** New-onset severe headache, visual disturbances, seizures, or altered consciousness in a patient with SCD, especially if accompanied by elevated blood pressure, should prompt consideration of PRES. Immediate neuroimaging and blood pressure evaluation are critical. Recognizing PRES is important because, unlike an infarct, the changes can be reversed with timely treatment, which can prevent permanent neurological damage.

#### 3.1.5. Acute Soft Head Syndrome

- **Definition:** an exceedingly rare neurological manifestation in SCD characterized by skull osteolysis and swelling [25];
- **Key Case Findings:** Mentioned as an exceedingly rare manifestation;
- D**iagnostic Challenges:** The diagnostic challenges regarding this syndrome are likely due to its rarity and potentially non-specific initial symptoms (headache, skull tenderness).
- **Pathophysiologic rationale:** Acute soft head syndrome is thought to be related to bone infarction in the skull;
- **Recommended diagnostic tests:** Skull imaging (X-ray, CT, MRI) to demonstrate osteolytic lesions is recommended;
- **Management Strategies and Outcomes:** Management for this syndrome is likely supportive, potentially involving pain control and addressing underlying crisis (limited specific guidance is available in the provided literature);
- **Clinical takeaway:** Acute soft head syndrome is an extremely rare neurological event in SCD; clinicians should be aware of its existence although specific guidance is limited in the provided literature.

### 3.2. Hematologic Complications

In addition to the well-known hematologic issues in SCD (such as aplastic crises or hyperhemolysis), there are rare syndromes of marrow or immune dysregulation that can be life-threatening. These often present with features that overlap with features of SCD crises and severe infection or inflammation, making diagnosis challenging.

#### 3.2.1. Acute Leukemia (Particularly AML)

(**Note:** *Acute leukemia is not a direct complication of SCD per se, but patients with SCD can develop leukemia, and there is debate about whether hydroxyurea or chronic marrow stress increases this risk. We include it here due to its rarity and diagnostic complexity in the SCD setting*.)

- **Definition:** malignant proliferation of myeloid blasts;
- **Key case findings:** An extensive review and case report published in 2023 identified over 50 published cases of acute leukemia in patients with SCD since 2000, often presenting with myelodysplastic changes and genetic abnormalities (e.g., chromosome 5/7 aberrations, TP53 mutations) [26]. The risk appears elevated, particularly in adults with severe disease or those exposed to certain therapies like hydroxyurea (though this association remains debated);
- **Diagnostic challenges:** The presentation of acute leukemia in patient with SCD can be mistaken for an aplastic crisis or severe sequestration crisis, since both can cause abrupt cytopenias. Fever and bone pain may be attributed to VOC or osteomyelitis. Diagnostic overshadowing occurs if clinicians assume all hematologic abnormalities are “just SCD complications.” A bone marrow biopsy (definitive test for leukemia) might be delayed if one is treating presumed parvovirus aplastic crisis, for example;
- **Pathophysiologic rationale:** Chronic inflammation and hematopoietic stem cell stress secondary to lifelong SCD pathology and prior treatments are thought to contribute to leukemogenic risk;
- **Recommended diagnostic tests:** Peripheral blood smear review, flow cytometry, bone marrow aspiration, and biopsy with cytogenetics and molecular testing are necessary for diagnosis of acute leukemia;
- **Management strategies and outcomes:** Management of acute leukemia follows standard leukemia protocols, but outcomes may be influenced by the underlying SCD and prior organ damage. Patients post-transplant may be at higher risk. The prognosis for AML in SCD is generally considered poor [26];
- **Prevention:** There is no established method of prevention for leukemia. While hydroxyurea’s leukemogenic risk is debated, the current evidence suggests it is low; nevertheless, regular blood-count monitoring in patients with SCD is standard and may catch early blast cells;
- **Clinical takeaway:** Acute leukemia is an emerging, rare complication in SCD; persistent or unexplained cytopenias warrant investigation, especially in those with severe disease or prior treatment exposures.

#### 3.2.2. Extramedullary Hematopoiesis (EMH)

- **Definition:** hematopoietic tissue expanding outside the bone marrow, often in response to chronic marrow dysfunction.
- **Key case findings:** EMH is a rarely described complication in adult patients with SCD. A 2024 case series and review identified varied localizations including localizations that were paravertebral, peri-articular in the hip, adrenal, hepatic, and splenic [27]. EMH was reported in a patient who presented with right-sided thoracic pain and was subsequently found to have a right adrenal mass [28];
- **Diagnostic challenges**: The symptoms depend on the localization (e.g., spinal cord compression from paravertebral masses, pain from peri-articular involvement, adrenal insufficiency, hepatic dysfunction, mass effect). These can be mistaken for VOC or other organ-specific complications;
- **Pathophysiologic rationale:** Chronic ineffective erythropoiesis in SCD drives increased hematopoietic activity, leading to tissue expansion in extramedullary sites [28];
- **Recommended diagnostic tests:** Diagnosis of EMH is established by histology and/or magnetic resonance imaging (MRI);
- **Management strategies and outcomes:** Management of EMH is noted as non-consensual, which reflects the rarity and varied presentations. Approaches to its management may include hydroxyurea optimization, transfusion therapy to suppress erythropoiesis, or radiation depending on the site and symptoms [27]. Surgical decompression may be needed for symptomatic spinal EMH;
- **Clinical takeaway:** EMH is a rare, location-variable complication in adult SCD; imaging (MRI) and, potentially, biopsy are key to diagnosis in patients with unexplained masses or symptoms referable to potential EMH sites.

#### 3.2.3. Hemophagocytic Lymphohistiocytosis (HLH)

- **Definition:** a life-threatening syndrome of immune dysregulation characterized by uncontrolled inflammation and tissue infiltration by activated lymphocytes and macrophages;
- **Key case findings:** HLH is a rare but severe complication in SCD, often triggered by infections (particularly viral). Its diagnosis can be challenging due to overlapping features with severe SCD crisis and infection [29]. A case report describes secondary HLH following coinfection with hepatitis A and E viruses in a child with sickle cell anemia [30];
- **Diagnostic challenges:** Distinguishing HLH from an acute SCD crisis with infection is challenging. Both can cause fever, organomegaly, cytopenias, high ferritin, and elevated liver enzymes. Physicians may treat presumed sepsis or acute chest syndrome while the HLH diagnosis is missed. A high ferritin level can be a clue, but sickle cell by itself (especially with liver involvement) can raise ferritin levels significantly. Notably, HLH typically involves extreme hyperferritinemia. While the HLH diagnostic criterion is ferritin > 500 ng/mL, in practice, HLH often shows ferritin levels in the many thousands; values > 10,000 ng/mL are highly suggestive of HLH [29,31]. Diagnostic overshadowing can occur if these signs are blamed on a sickle cell liver/splenic sequestration or severe infection alone, delaying the critical immunologic treatment for HLH;
- **Pathophysiologic rationale:** Triggering events like infection or severe crisis lead to uncontrolled activation of cytotoxic T cells and macrophages, resulting in a cytokine storm and organ damage;
- **Recommended diagnostic tests:** Evaluation of HLH is based on HLH diagnostic criteria, which include laboratory tests (CBC, liver function, triglycerides, fibrinogen, ferritin, sCD25) and bone marrow biopsy for hemophagocytosis. Identifying the underlying trigger is crucial [29,30];
- **Management strategies and outcomes:** Treatment of HLH involves addressing the underlying trigger (e.g., treating infection) and immune suppression using chemotherapy regimens developed for HLH (e.g., etoposide-containing protocols) [29]. The outcomes depend on timely diagnosis and treatment of the trigger and HLH itself;
- **Prevention:** Preventing HLH in SCD is not straightforward, as it often depends on unpredictable infections. However, one can argue that preventing severe infections through vaccination, penicillin prophylaxis (in children), and prompt treatment of infections could reduce the trigger risk. Also, rapidly controlling severe sickle crises and avoiding transfusional iron overload (iron can fuel macrophages) might theoretically help. Clinicians should maintain a low threshold for screening for HLH in any patient with SCD with atypical severity of illness (e.g., lab signs of hyperinflammation), which in effect is secondary prevention (catching early);
- **Clinical takeaway:** Unexplained fever, worsening cytopenias, organ dysfunction, and significantly elevated ferritin in a patient with severe SCD or infection should prompt evaluation for HLH; timely diagnosis and immune-modulatory therapy are critical.

### 3.3. Cardiopulmonary and Vaso-Occlusive Complications (Severe Forms)

While acute chest syndrome and pulmonary hypertension are common, certain cardiopulmonary events or severe vaso-occlusive phenomena constitute rare, life-threatening complications. They often involve extensive bone marrow necrosis with systemic embolization or simultaneous failure of multiple systems.

#### Fat Embolism Syndrome (FES) and Severe Bone Marrow Necrosis

- **Definition:** FES is a clinical syndrome caused by systemic embolization of fat globules, typically from fractured bones. In SCD, it results from extensive bone marrow necrosis and subsequent embolization of marrow contents [32]. Bone marrow necrosis itself is a rare but serious complication [33];
- **Key case findings:** FES and severe bone marrow necrosis are among the most severe and rare SCD complications. The case reports and reviews that discuss FES in SCD discuss ~80+ reported cases of FES in SCD. Notably, FES in SCD, paradoxically, appears more common in patients with milder genotypes (HbSC or HbSβ^+^ thalassemia) who have had relatively few prior crises [32,34]. Its clinical presentation typically involves a seeming severe pain crisis followed within 1–3 days by acute respiratory failure, neurological changes (ranging from confusion to coma), fever, and, often, a new petechial rash. Laboratory clues include a sudden drop in levels of hemoglobin and platelets. Some patients have evidence of recent parvovirus B19 infection as a trigger for marrow necrosis. One unusual presentation of previously undiagnosed sickle-β+ thalassemia involved FES [35]. Bone marrow necrosis is associated with high morbidity and mortality [36]. Cerebral fat embolism syndrome without myonecrosis has also been reported [37];
- **Diagnostic challenges:** Clinical features of FES (neurological changes, respiratory distress, petechial rash) can overlap with severe acute chest syndrome, stroke, or MOF [32]. Its diagnosis requires a high index of suspicion in the context of severe bone pain or evidence of bone marrow infarction. Characteristic MRI findings like the “starfield” pattern in the brain can support the diagnosis of cerebral fat embolism [38]. Diagnostic overshadowing is a big risk here, as it result from attributing the multi-system deterioration to “severe sickle cell crisis” or sepsis rather than recognizing fat embolism. A clue can be the degree of anemia and reticulocytopenia being out of proportion to that of usual VOC, which suggests bone marrow necrosis;
- **Pathophysiologic rationale:** Extensive sickling in the bone marrow causes infarction and necrosis of marrow tissue. This necrotic marrow releases fat globules (and pro-inflammatory substances) into venous blood. These fat emboli lodge in pulmonary capillaries (causing acute respiratory distress similar to ARDS) and can traverse to systemic circulation (especially if there is a right-to-left shunt or via overwhelmed pulmonary capillaries), causing cerebral emboli and other organ damage. Having an inflammatory response to the fat leads to the fever and systemic inflammatory response [32];
- **Recommended diagnostic tests**: Imaging of bones may show infarction. Pulmonary function tests and imaging (CT scan) may show evidence of lung injury. Neurological assessment and imaging (MRI, particularly with diffusion-weighted sequences for a “starfield” pattern) are important. Detection of fat globules in urine, sputum, or bronchoalveolar lavage can support the diagnosis but is not always sensitive or specific [32];
- **Management strategies and outcomes:** Immediate ICU care is required. Supportive care for FES includes high-flow oxygen or mechanical ventilation for respiratory failure, and often invasive monitoring. Exchange transfusion is widely recommended as soon as FES is suspected, to rapidly reduce the proportion of sickled cells and halt ongoing infarction. Evidence suggests that prompt red cell exchange improves survival [32];
- **Prevention:** Preventing FES is challenging because it often strikes unexpectedly in a patient who was not previously considered high-risk. However, one suggestion is to ensure aggressive management of any very severe pain crisis (some have advocated for early exchange transfusion in an unusually severe VOC to preempt marrow necrosis) [39];
- **Clinical takeaway:** Severe bone pain followed by rapid neurological or respiratory deterioration should prompt consideration of bone marrow necrosis and FES. This is a medical emergency—intensive supportive care and urgent exchange transfusion can be life-saving. Even with optimal care, FES carries a high mortality rate, so maintaining vigilance for this complication in any unusually severe SCD crisis is essential;
- **Acute chest syndrome (ACS) vs. FES—a brief note:** It is worth noting that FES can be mistaken for ACS (and ACS is common in SCD). The presence of neurological symptoms or severe cytopenias can help differentiate the two. Some FES cases were likely labeled as “atypical ACS” historically. In practice, when a patient with SCD presents with ACS but has unusually severe systemic findings, clinicians should consider FES on the differential and manage accordingly (with exchange transfusion and broader supportive care).

### 3.4. Abdominal Complications

#### Hepatic Complications: Sequestration, Cholestasis, and Liver Transplantation

- **Definition:** a spectrum of liver dysfunction in SCD, beyond typical sickling hepatopathy, including acute hepatic sequestration (sudden trapping of red cells in the liver), sickle cell intrahepatic cholestasis (severe cholestasis due to sinusoid occlusion), and chronic liver damage, that sometimes requires transplantation;
- **Key case findings:** Sickle cell hepatopathy is a well-recognized complication, but acute hepatic sequestration crisis is considered rare [40,41]. Sickle cell intrahepatic cholestasis is extremely rare but often fatal [42]. Reviews cover the liver in SCD and management of complications [43]. Liver transplantation has been performed in patients with SCD, sometimes after hematopoietic stem cell transplant (HSCT) for the underlying SCD [44];
- **Diagnostic challenges:** Acute abdominal pain, jaundice, and elevated liver enzymes are common in various SCD crises. Distinguishing acute hepatic sequestration (rapid drop in hemoglobin, tender hepatomegaly) from a vaso-occlusive crisis or cholecystitis/cholangitis requires careful assessment [41]. Intrahepatic cholestasis presents with profound jaundice, coagulopathy, and synthetic dysfunction [42];
- **Pathophysiologic rationale:** Vaso-occlusion in the hepatic microcirculation leads to ischemia, sickling, red cell trapping, inflammation, and progressive liver damage [40]. Intrahepatic cholestasis involves extensive occlusion of hepatic sinusoids [42];
- **Recommended diagnostic tests:** The recommended tests for hepatic complications include liver function tests (ALT, AST, bilirubin, alkaline phosphatase), a coagulation panel, and ultrasound/CT of the abdomen to assess the liver size and look for other causes (gallstones, common bile duct dilation). Liver biopsy may be needed for unclear cases or suspected cholestasis [40,41,42,43];
- **Management strategies and outcomes:** Management of hepatic complications depends on the specific complication. Acute hepatic sequestration may require exchange transfusion [41]. Intrahepatic cholestasis is medically challenging; supportive care and exchange transfusion are options, but their prognosis is poor [42]. Liver transplantation is a consideration for end-stage liver disease, potentially combined with or following HSCT [44];
- **Prevention:** To prevent hepatic sequestration or cholestasis, one can only generalize: avoid dehydration and hypoxia, which precipitate sickling, and treat the sickle cell disease aggressively to reduce crisis frequency (hydroxyurea, transfusions for severe phenotype). Prompt transfusion in the case of any acute hepatic involvement might prevent progression to full cholestatic syndrome. Closely monitoring patients with known liver iron overload or hepatitis during crises is warranted since they may tolerate less hepatic stress;
- **Clinical takeaway:** Severe or rapidly worsening liver function abnormalities, significant hepatomegaly, or profound jaundice in patient with SCD warrants investigation for acute hepatic sequestration or intrahepatic cholestasis; these are serious complications that require specialized management.

### 3.5. Musculoskeletal Complications

A rare musculoskeletal complication is deep compartment syndrome without myonecrosis, representing an atypical presentation.

#### Deep Compartment Syndrome Without Myonecrosis

- **Definition:** increased pressure within a muscle compartment, compromising circulation and tissue function, but without the extensive muscle tissue death typical of myonecrosis;
- **Key case findings:** Deep compartment syndrome is a rare complication of SCD, often occurring in extremities. A case report describes deep compartment syndrome without myonecrosis in patient with SCD [45];
- **Diagnostic challenges:** Pain and swelling in an extremity can mimic typical vaso-occlusive crisis. Diagnosis of this syndrome requires clinical suspicion based on the severity and location of pain, the tenseness of the compartment, and, potentially, the measurement of compartment pressures;
- **Pathophysiologic rationale:** The pathophysiologic rationale includes vaso-occlusion leading to tissue swelling within a closed fascial compartment. While bone infarction is common, severe soft tissue edema leading to compartment syndrome is less frequent;
- **Recommended diagnostic tests:** Clinical examination is a recommended test for the diagnosis of this syndrome. Measurement of compartment pressures can confirm the diagnosis. MRI may show muscle edema;
- **Management strategies and outcomes**: Urgent fasciotomy may be required to relieve pressure and preserve the tissue’s viability [45]. Prompt intervention is key to preventing permanent damage;
- **Clinical takeaway:** Severe, disproportionate, and localized pain and swelling in an extremity with signs of tense compartment in patient with SCD should raise suspicion for compartment syndrome, even without apparent muscle death; urgent surgical consultation is needed.

### 3.6. Dermatologic Complications

Cutaneous manifestations are not traditionally the focus of acute SCD care, but they can significantly affect patients’ quality of life and may be overlooked. Chronic leg ulcers are the most recognized dermatologic complication of SCD, although they are not exceedingly rare (affecting ~2.5–10% of adults with HbSS) [46]. However, they illustrate how SCD’s vasculopathy can involve the skin. Rarely, other dermatologic issues have been noted, including unusual infections and vasculitic lesions.

- **Leg Ulcers:** Often occurring around the ankles, these chronic, refractory ulcers are believed to result from microvascular occlusion, local trauma, and perhaps impaired wound healing due to hemolysis-related endothelial dysfunction. They can be very painful and lead to recurrent infections. Although not “rare” in the global SCD population, they are underrepresented in the literature relative to their impact. Their management is challenging (wound care, skin grafts, transfusions, and maybe hyperbaric oxygen) [47]. Their prevention revolves around avoiding trauma, using compression/support stockings to improve circulation, and using hydroxyurea, which has been associated with promoting ulcer healing in some cases;
- **Hydroxyurea-related skin ulcers:** A subset of patients on hydroxyurea develop painful leg ulcers as a side effect of the drug. This is relatively uncommon but important to distinguish from SCD-related ulcers, as the management may involve reducing the dose or switching to a different therapy [48];
- **Livedoid vasculopathy:** Recent reports describe patients with SCD with lesions consistent with livedoid vasculopathy (a thrombotic skin condition that causes painful ulcers on the legs) [47];
- **Infections:** Patients with SCD can get unusual skin infections due to their immune-compromised state (asplenia) and frequent hospital exposures. Chronic ulcers can be colonized with atypical organisms (including pseudomonas, atypical mycobacteria) and fungal infections have been noted anecdotally [49];
- **Others:** There is evidence that patients with SCD have a lower incidence of cutaneous malignancies (like melanoma and non-melanoma skin cancer) than expected, possibly due to their shorter lifespan historically or protective factors of hemolysis (this is an evolving area) [50]. Also noted are pigmentary changes (hyperpigmentation of skin and nails) in children on chronic transfusions or hydroxyurea [51].

A summary of key rare complications and their clinical features, diagnostic indicators, recommended investigations, treatment considerations, and prognostic implications is provided in Table 1.

## 4. Discussion

The diagnostic challenges highlighted in this review underscore a pervasive pattern in the care of patients with sickle cell disease: the insidious impact of diagnostic overshadowing and resultant diagnostic inertia. Clinicians, appropriately primed to recognize the frequent manifestations of SCD, may inadvertently attribute severe or atypical symptoms solely to common vaso-occlusive pain, acute chest syndrome, or anemia. This phenomenon is evident across various systems; for instance, severe back pain is often ascribed to bone crisis rather than prompting urgent spinal imaging for epidural hematoma, and neurological deficits occurring in the context of systemic illness are not fully investigated for atypical infarcts [7,12,13] or the involvement of multiorgan failure [15]. Similarly, persistent cytopenia may be dismissed as baseline effects of SCD instead of triggering evaluation for acute leukemia [26] or HLH [29]. Unexplained masses or organ dysfunction might not immediately suggest extramedullary hematopoiesis [27] or specific hepatic complications [27,40,41,42].

From a cognitive science perspective, diagnostic overshadowing reflects a failure to recalibrate differential diagnoses in light of atypical presentations, and it is exacerbated by high clinical workloads and systemic pressures such as emergency department crowding and time limitations [52]. Moreover, the complex multisystem involvement inherent to SCD, compounded by overlapping symptomatology, increases the risk of clinical complacency.

The consequences of this diagnostic inertia are significant and often severe. Delayed diagnosis prevents timely, specific interventions. For conditions like spontaneous epidural hematoma or atypical brain/spinal infarcts, prompt imaging and intervention are critical for preserving patients’ neurological function. Acute leukemia in SCD, often presenting with high-risk features, requires urgent diagnosis and initiation of complex treatment protocols; delay can worsen prognosis. Severe complications like acute multiorgan failure syndrome and fat embolism syndrome carry high mortality rates; delayed recognition and intensive support likely contribute to poor outcomes.

Practical strategies to combat diagnostic overshadowing and improve frontline clinical care for rare SCD complications include the following:

- Maintaining a high index of suspicion: Clinicians must consciously consider rare possibilities when a patient’s symptoms are severe, persistent, or atypical for common SCD events, especially when symptoms do not respond to standard management;
- Structured differential diagnosis: Develop and utilize differential diagnosis frameworks that explicitly include rare SCD complications alongside common ones based on symptom clusters. The emergency department represents a critical juncture where atypical presentations must be promptly recognized [53];
- Prompt and appropriate investigations: Do not hesitate to order specific diagnostic tests (e.g., urgent MRI for neurological/spinal symptoms, bone marrow biopsy for unexplained cytopenia or suspected HLH, cross-sectional imaging for unexplained masses or specific abdominal complaints, urgent ophthalmologic evaluation for vision changes, compartment pressure measurement for suspected compartment syndrome when initial clinical assessment raises suspicion for a rare entity, even in the setting of a typical-appearing sickle crisis);
- Leveraging multidisciplinary expertise: Engage specialists (e.g., neurology, neurosurgery, oncology, radiology, transfusion medicine, gastroenterology, ophthalmology, orthopedics) early when a rare complication is suspected;
- Education and awareness: Continually educate healthcare providers across all levels of care, particularly those in emergency departments and general hospital wards, about the spectrum of rare SCD complications and the pitfalls of diagnostic overshadowing.

Collectively, these strategies hold promise for reducing diagnostic inertia, improving patient outcomes, and advancing the standard of care in this complex population.

Future directions to enhance understanding and management of these rare events could involve the following:

- Centralized reporting systems: Establishing a dedicated national or international platform for reporting rare SCD complications beyond individual case reports would create a larger dataset for analysis;
- SCD registries: Expanding existing SCD registries to include detailed documentation of rare complications would allow for better estimation of incidence, identification of risk factors, evaluation of management strategies, and assessment of outcomes;
- App-based decision support tools: Developing digital tools or algorithms integrated into electronic health records or standalone applications could prompt clinicians to consider specific rare diagnoses based on present symptom constellations, guiding appropriate investigations and initial management.

Addressing rare complications requires moving beyond anecdotal experience towards more systematic data collection and integrating this knowledge into routine clinical practice through increased awareness and structured diagnostic approaches.

## 5. Implications for Practice

Building upon the above strategies, there are broader steps the healthcare community can take. One is to integrate decision support into electronic medical records for SCD management, and another is the use of alerts or order set suggestions when certain combinations of symptoms are charted (e.g., “Consider checking ferritin for fever + cytopenia in SCD” as a prompt). Another is establishing direct communication pathways; for example, emergency physicians in regions with many patients with SCD might have an on-call hematologist to discuss difficult cases in real time. This fosters a culture where asking “Could it be something unusual?” becomes routine rather than rare.

## 6. Future Directions and Research

Advancing our understanding and management of rare SCD complications will require concerted efforts beyond individual case reports. There is a clear need for a centralized repository of rare SCD complication cases. We propose establishing dedicated national or international registries, potentially under the auspices of organizations, to collect data on occurrences of these rare events. Such a registry would allow aggregation of sufficient case numbers to estimate incidence, identify risk factors (e.g., why HbSC patients appear to get FES more), and compare management outcomes. For instance, a registry could prospectively document all cases of HLH in SCD across centers, which would lead to better understanding and perhaps tailored treatment recommendations. Similarly, an international collaboration (leveraging African, Middle Eastern, and Indian subcontinent data where the SCD burden is high) could shed light on how frequently these events happen in under-studied populations and what the outcomes are. This systematic data collection moves us beyond the use anecdotal evidence.

## 7. Limitations

Despite employing a comprehensive literature search and synthesis, this review has inherent limitations. First, there is likely publication bias, as dramatic cases (often with poor outcomes or novel presentations) are more likely to be published than routine or mild occurrences. This skews our perception of these complications to the severe end and may overestimate their mortality. Conversely, underreporting is a concern: rare complications in resource-limited settings or subtle cases that were resolved might never enter the literature, which could lead to underestimation of true incidence. Second, our review included only English-language publications and may have missed non-English case reports, which could be significant, especially considering the regions where SCD is prevalent. Third, the quality of evidence is low; we rely on case series and reports without control groups. Finally, our narrative approach, while broad, had to be selective in focus. We emphasized acute, life-threatening complications; other rare issues (like chronic kidney complications beyond the scope of this paper, or mild dermatologic phenomena) were not exhaustively reviewed.

These limitations highlight the need for further research and cautious interpretation of our conclusions. Clinicians should use our synthesis as a guide but remain critical and tailor their decisions to individual patient contexts.

## 8. Conclusions

Rare and unusual complications, although infrequent, represent significant potential sources of morbidity and mortality in patients with sickle cell disease. These entities, ranging from spontaneous epidural and subdural hematoma and atypical infarcts to acute leukemia, severe multiorgan failure syndrome, hemophagocytic lymphohistiocytosis, localized extramedullary hematopoiesis, severe hepatic complications, and compartment syndrome, necessitate a level of vigilance beyond routine management of common crises. The phenomenon of diagnostic overshadowing poses a substantial barrier to timely recognition. Clinicians caring for patients with SCD must cultivate a high index of suspicion and employ structured diagnostic thinking when confronted with atypical or disproportionately severe presentations. Prompt application of appropriate investigations, such as advanced imaging or specific laboratory testing, is critical for differentiating rare complications from common ones and facilitating effective intervention. The current evidence base, largely derived from case reports, highlights the need for enhanced knowledge dissemination and collaborative data collection. Further study, including the development of prospective registries and multi-institutional reporting platforms, is essential to better understand the incidence, risk factors, optimal management, and long-term outcomes of these rare complications. Increased awareness and a proactive diagnostic approach are paramount to improving the care and prognosis for patients experiencing the infrequent but severe manifestations of SCD.

## Figures and Tables

**Table 1:** Summary of rare complications in sickle cell disease.

Rare Complication	Clinical Presentation	Diagnostic Clue	Recommended Action	Prognostic Implications
**Neurological Complications**				
Spontaneous Epidural Hematoma	Sudden onset back pain, radicular pain, neurological deficits (weakness, sensory changes, bowel/bladder dysfunction). Frequently during sickle crises.	Unexplained or disproportionately severe spinal pain or neurological deficits in a patient with SCD. Non-traumatic presentation.	Urgent MRI of the affected spine or brain. Neurosurgical intervention critical for decompression.	Favorable if diagnosed and treated promptly; delayed treatment risks permanent neurological deficits.
Spontaneous Subdural Hematoma	Headache, neurological changes, papilledema. Can mimic dural sinus thrombosis.	Atypical headaches or neurological signs, especially with new papilledema.	Urgent MRI or CT brain. Neurosurgical evaluation for management.	Potentially life-threatening; early diagnosis improves outcomes but morbidity can be high.
Brain and Spinal Infarcts	Neurological impairment in unusual locations (e.g., supra/infratentorial, anterior spinal cord) or contexts (e.g., during septic shock, MOF).	Severe or atypical neurological deficits, even with systemic illness. Recognizing infarcts in unusual locations.	Brain and spinal MRI. Evaluate for infection, antiphospholipid antibodies. Treat underlying crisis/infection. Exchange transfusion to keep HbS < 30%. Consult neurology/Nneurosurgery.	Variable; early intervention can improve neurological recovery; delayed diagnosis may result in permanent deficits.
**Hematologic Complications**				
Acute Leukemia (AML)	Atypical or persistent cytopenias; overlap with severe aplastic or sequestration crises; fever, fatigue, bleeding, infection.	Persistent atypical cytopenias in patients with SCD, especially with prior severe disease.	Peripheral blood smear, flow cytometry, bone marrow biopsy with cytogenetics and molecular testing. Oncology/hematology consult urgent.	Poor prognosis; delayed diagnosis worsens outcomes; aggressive treatment necessary.
Hemophagocytic Lymphohistiocytosis (HLH)	Persistent fever, worsening cytopenias, splenomegaly, hyperferritinemia, coagulopathy, organ dysfunction; overlaps with severe infection/crisis.	Unexplained fever, worsening cytopenias, very high ferritin (>500 ng/mL), immune activation signs.	Evaluate HLH criteria; labs: CBC, LFTs, coags, triglycerides, fibrinogen, ferritin, sCD25; bone marrow biopsy. Treat triggers; immunosuppression; urgent Hematology/Oncology/Critical Care consult.	High mortality if untreated; early diagnosis improves survival.
**Cardiopulmonary and Vaso-occlusive Complications (Severe Forms)**				
Acute Multiorgan Failure (MOF) Syndrome	Acute failure in ≥2 vital organs during acute VOC or progressive acute chest syndrome. Rapid progression.	Rapidly progressive multiorgan dysfunction without other MOF causes.	Organ function evaluation, imaging (CXR/CT), blood cultures, assess for bone marrow necrosis/fat embolism. Intensive supportive care; consider urgent exchange transfusion.	Very high mortality; early aggressive treatment required.
Fat Embolism Syndrome (FES)/Severe Bone Marrow Necrosis	Abrupt neurologic deterioration, respiratory failure, multiorgan dysfunction; petechial rash possible; may occur with unusual presentations (e.g., de novo diagnosis).	Severe bone pain followed by rapid neuro/respiratory decline; “starfield” pattern on brain MRI.	Supportive care including respiratory support and pain control; exchange transfusion; critical care and hematology consultation. Bone imaging, chest CT, brain MRI. High mortality risk.	High mortality risk; prognosis poor without timely intervention.
**Abdominal Complications**				
Hepatic Sequestration Crisis	Acute right upper quadrant pain, tender hepatomegaly, rapid hemoglobin drop.	Tender hepatomegaly with acute anemia and elevated liver enzymes.	Supportive care, pain management, consider exchange transfusion. Gastroenterology/hematology consult.	Generally favorable with prompt treatment; risk of severe anemia and shock if untreated.
Sickle Cell Intrahepatic Cholestasis	Profound jaundice, liver dysfunction (coagulopathy, synthetic failure).	Severe jaundice with direct hyperbilirubinemia, coagulopathy.	Intensive supportive care; may require exchange transfusion; liver biopsy for confirmation. Critical care needed.	High mortality if diagnosis delayed; early treatment essential.
Extramedullary Hematopoiesis (Adrenal, Hepatic, Splenic)	Symptoms depend on specific site: adrenal insufficiency, hepatic dysfunction, mass effect.	Unexplained masses or symptoms referable to these sites.	Imaging (MRI), possibly biopsy; management varies including observation, hydroxyurea, transfusion, radiation.	Variable prognosis depending on site and severity.

## Data Availability

No new data were created or analyzed in this study.
